# The Fungal Cell Death Regulator *czt-1* Is Allelic to *acr-3*

**DOI:** 10.3390/jof5040114

**Published:** 2019-12-06

**Authors:** A. Pedro Gonçalves, Kevin McCluskey, N. Louise Glass, Arnaldo Videira

**Affiliations:** 1 ICBAS-Instituto de Ciências Biomédicas Abel Salazar, University of Porto, Rua de Jorge Viterbo Ferreira 228, 4050-313 Porto, Portugal; 2 Fungal Genetics Stock Center, Department of Plant Pathology, Kansas State University, 4024 Throckmorton Plant Sciences Center, Manhattan, KS 66506, USA; 3 Department of Plant and Microbial Biology, University of California, Berkeley, CA 94720, USA; 4 Environmental Genomics and Systems Biology Division, Lawrence Berkeley National Laboratory, 1 Cyclotron Road, Berkeley, CA 94720, USA; 5 i3S—Institute for Research and Innovation in Health, University of Porto, Rua Alfredo Allen 208, 4200-135 Porto, Portugal

**Keywords:** antimicrobial drug resistance, cell death, CZT-1, ABC-3, acriflavine, staurosporine

## Abstract

Fungal infections have far-reaching implications that range from severe human disease to a panoply of disruptive agricultural and ecological effects, making it imperative to identify and understand the molecular pathways governing the response to antifungal compounds. In this context, CZT-1 (cell death-activated zinc cluster transcription factor) functions as a master regulator of cell death and drug susceptibility in *Neurospora crassa*. Here we provide evidence indicating that *czt-1* is allelic to *acr-3*, a previously described locus that we now found to harbor a point mutation in its coding sequence. This nonsynonymous amino acid substitution in a low complexity region of CZT-1/ACR-3 caused a robust gain-of-function that led to reduced sensitivity to acriflavine and staurosporine, and increased expression of the drug efflux pump *abc-3*. Thus, accumulating evidence shows that CZT-1 is an important broad regulator of the cellular response to various antifungal compounds that appear to share common molecular targets.

## 1. Introduction

Human fungal infections have been, until recently, a largely underestimated public health problem with dramatic worldwide ramifications. Superficial skin infections occur in approximately a quarter of the world’s population, and invasive fungal infections, while less incident, are associated with high mortality rates—about 1.5 million people every year [1]. Fungal disease of plant and livestock has been leading to major social and economic costs in food systems and driving several animal species to extinction [2,3]. In addition, fungal infections are on the rise due to climate change-associated global warming [2,3].

Theoretically, most human fungal infections are relatively easy to treat provided that access to antifungal drugs is assured. However, a restricted number of drug options against fungal infections are available, including polyene (amphotericin B), echinocandin and azole drugs [4,5]. The success of available treatments is hampered by the development of antifungal drug resistance that can involve target modification, overexpression of efflux pump-encoding genes or hot spot amino acid substitutions [4,5]. The induction of regulated forms of cell death is a common attribute of antifungal drugs and, therefore, understanding the molecular players and pathways regulating these cellular processes is of vital importance for the identification of suitable drug targets [6]. In order to achieve this goal, fungal models, such as the ascomycete *Neurospora crassa*, are of great importance due to the wide availability of genetic tools, such as targeted gene deletion strain collections and the accumulation of strains obtained over the years by classical forward genetics methodologies [7].

Staurosporine is a natural bacterial alkaloid, protein kinase inhibitor and prototypical anticancer drug that has been customarily used as a cell death inducer in various organisms, including fungi [6]. The *N. crassa* response to staurosporine involves well-defined alterations in the levels of cytosolic calcium, a process mediated by a putative transient receptor potential (TRP) channel at the plasma membrane and regulated by phospholipase C [8], as well as reactive oxygen species-dependent signaling [9]. At the transcriptional level, the *N. crassa* response to staurosporine is largely controlled by the Zn_2_Cys_6_ binuclear cluster transcription factor CZT-1 [10]. Here we report that *czt-1* is allelic to *acr-3*, a locus that had been described in a mutant *N. crassa* strain obtained decades ago by random mutagenesis and that exhibits increased resistance to the fungal growth inhibitors acriflavine and malachite green [11]. We show that a single amino acid change in *czt-1* results in gain-of-function and is the likely cause for the enhanced tolerance to acriflavine of the *acr-3* strain. This report expands the importance of CZT-1 as an important regulator of susceptibility to antifungal compounds.

## 2. Materials and Methods

Routine cultivation of *N. crassa* was performed on Vogel’s minimal medium with 2% sucrose (plus 1.5% agar for solid medium). Asexual spores were obtained by growing strains in glass tubes with slanted medium for one week until full sporulation was evident. Mutant strains were obtained from the Fungal Genetics Stock Center [7]. The genomic sequence of NCU09974 in the *acr-3* mutant was obtained using a routine sequencing methodology [12]. Radial growth and spot assays were conducted as previously described [10]. Staurosporine was obtained from LC Laboratories and acriflavine and malachite green from Sigma-Aldrich. For quantitative real-time PCR experiments (qRT-PCR), asexual spores at a concentration of 10^6^ cells/mL were grown at 26 °C in liquid VMM for 7 h; RNA was then isolated using the ZR Fungal/Bacterial RNA MicroPrep kit (Zymo Research), used for cDNA preparation using the SuperScript First-Strand Synthesis System kit (Life Technologies) and the relative expression of *abc-3* in different strains using *actin* (NCU04173) as the reference gene was obtained using the 2^−ΔΔ*C*t^ calculation method from mixes containing previously described primers [13] and SYBR Green (Bio-Rad) that were analyzed in a Corbett Research Rotor-Gene 6000 thermocycler. Statistical analysis of qRT-PCR results was conducted on Prism 6 software (Graphpad).

## 3. Results and Discussion

The application of classical forward genetics methodologies in *N. crassa* has resulted in a collection of strains displaying morphological and developmental phenotypes that has been complemented with a genetic map of more than 1000 phenotypic markers and hundreds of other features like telomeres, centromeres, nucleolus organizer region, translocations, inversions and duplications [14]. More recently, whole genome resequencing has been used to identify the genome modifications underlying some of those phenotypes [15]. The *N. crassa* mutant FGSC1215 (*acr-3 mat-a*) was generated by UV light-based random mutagenesis and exhibits increased resistance to acriflavine [11]; *acr-3* (*acriflavine resistant-3*) is one of seven loci in *N. crassa* associated with altered tolerance to this drug [14,16]. Acriflavine (a mixture of 3,6-diamino-10-methylacridinum chloride (trypaflavine) and 3,6-diaminoacridine) is a heteroaromatic dye with antibacterial, antiviral, anti-inflammatory and anticancer effects [17].

**Figure 1:**
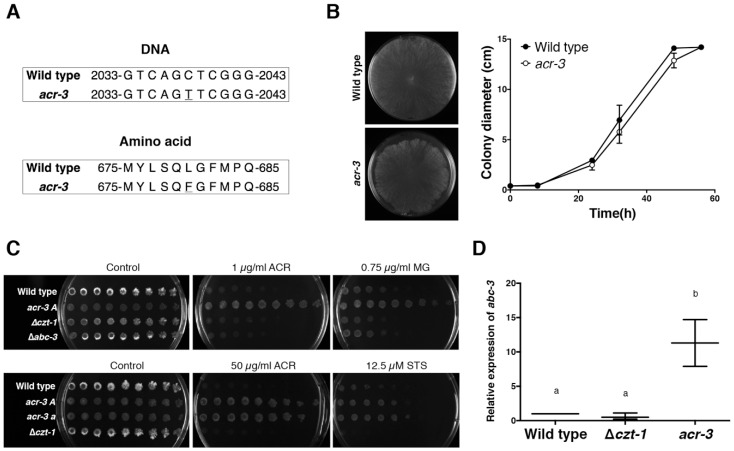
A point mutation in *czt-1* explains the increased tolerance of the *acr-3* mutant strain to acriflavine, malachite green and staurosporine. (**A**) DNA (top) and amino acid (bottom) sequence of a segment of *czt-1* in wild type and *acr-3* strains. A C>T (L>F) substitution was found in the *acr-3* strain (underlined). Numbering corresponds to the larger transcript of *czt-1*. (**B**) Growth on solid Vogel’s minimal medium of wild type and *acr-3* strains was followed across time. Images on the left were obtained 48 h after inoculation; on the right, colony diameter measurements. No statistically significant differences between the two strains were found. (**C**) Spot assays were performed to analyze the tolerance of strains labeled on the left to the indicated drugs. ACR, acriflavine; MG, malachite green; STS, staurosporine. (**D**) Relative expression of the *abc-3* gene as measured by qPCR using *actin* as the reference gene. ANOVA followed by a Tukey post-hoc test showed that the expression of *abc-3* in the *acr-3* strain (“b”) was significantly higher than in wild type or ∆*czt-1* (“a”); *p*-value < 0.008. Three independent experiments were performed.

The *acr-3* locus has been mapped to linkage group I of *N. crassa*, between *un-16* and *suc* [14,16]. We have previously evaluated the presence of SNPs in NCU09975, a gene located in this region that encodes ABC-3, a drug efflux pump and member of the ATP-binding cassette (ABC) transporter family [13]. However, no SNPs in *abc-3* were found in strains FGSC1215 (*acr-3 mat-a*), FGSC1209 (*acr-3 mat-A*) or FGSC875 (*acr-1 mat-a*) [18]. Thus, we hypothesized that another candidate gene that could play a role in resistance to acriflavine in the *acr-3* mutant was *czt-1*; *czt-1* is also located in the genomic region previously identified to contain the *acr-3* locus. In fact, *czt-1* and *abc-3* are adjacent to each other and CZT-1 is a transcriptional regulator of *abc-3* [10,13]. Targeted sequencing of *czt-1* (NCU09974) in FGSC1215 (*acr-3*) revealed a C>T non-synonymous mutation in its coding sequence (Figure 1A). Two transcripts or variants of *czt-1*, encoding proteins 780 and 685 amino acids long, respectively, have been annotated. The candidate mutation in *czt-1* corresponded to a leucine to phenylalanine change in position 680 in the larger CZT-1 variant or position 585 in the shorter protein variant. CZT-1 is a Zn_2_Cys_6_ binuclear cluster transcription factor and the candidate mutation was located in a low complexity region with polar amino acid compositional bias but not in any of the conserved and previously described “DNA-binding domain”, “fungal-specific transcription factor domain” or “uncharacterized transcriptional regulatory protein domain” [10]. It will be interesting to biochemically characterize the different regions of the CZT-1 protein in future studies.

The *acr-3* mutant grew slightly slower than the wild type (Figure 1B). We confirmed that the *acr-3* mutant displays resistance to acriflavine using a spot assay; acriflavine at a concentration of 1 µg/mL almost completely inhibited the growth of the wild type, while the *acr-3* mutant remained unaffected (Figure 1C). Strains where *czt-1* or *abc-3* were deleted showed similar sensitivity to acriflavine as the wild type parental cells (Figure 1C). The *acr-3* mutant was also more resistant to the antimicrobial malachite green, as previously reported [19], whereas a ∆*czt-1* mutant was slightly more sensitive than the wild type (Figure 1C). Our previous work showed that the deletion of *czt-1* results in high susceptibility to staurosporine [10]. Interestingly, *acr-3* mutants were more resistant to staurosporine than the wild type strain (Figure 1C). Thus, the previously reported genetic mapping of the *acr-3* allele that positioned the causative locus in the *czt-1*-*abc-3* region [14,16], together with the sequence analysis of the *acr-3* mutant strain showing a point mutation in *czt-1*, the drug susceptibility profiles of the ∆*czt-1* and *acr-3* strains, and the known function of CZT-1 as a drug tolerance regulator [10], all indicate that *acr-3* is allelic to *czt-1*.

Since the ∆*czt-1* deletion is a loss-of-function mutation, our comparison between *czt-1* and *acr-3* alleles suggested that the leucine to phenylalanine substitution in *acr-3* caused a gain-of-function phenotype. In line with this hypothesis, the expression of *abc-3*, a key target of CZT-1 [10], was upregulated in the *acr-3* strain (Figure 1D). ABC-3 exports staurosporine from the cytosol to the extracellular space [13] and could hypothetically function in a similar way with acriflavine. In addition to *abc-3*, CZT-1 regulates the expression of multiple ABC transporters, including azole resistance-associated *cdr-4* as well as *atrf* and *atrf-2* and others that lack functional characterization but contain typical ABC transporter domain features [10]. Thus, it appears plausible to speculate that the leucine to phenylalanine mutation in CZT-1/ACR-3 could result in an increase in the activity of one or more drug efflux pumps and, consequently, in increased tolerance to the antifungals acriflavine, staurosporine and malachite green. Our targeted gene sequencing approach does not allow us to rule out the possibility that potential mutations in other genes included in the range between *un-16* and *suc* could be relevant for the drug sensitivity profile of the *acr-3* strain, but our results suggest that the mutation found in the coding region of *czt-1* is likely causative of the drug tolerance profile of the *acr-3* mutant. Moreover, high doses of acriflavine (50 µg/mL) were almost ineffective against both mating type strains of *acr-3* (Figure 1C), indicating that the mutation not only leads to gain-of-function of the respective protein, but also that its effect is very robust. In line with our results, a number of previous studies have reported point mutations in genes encoding Zn_2_Cys_6_-family transcription factors that result in gain-of-function phenotypes [20,21].

In summary, we report here that the *acr-3* strain not only exhibits increased resistance to acriflavine and malachite green, but also to the archetypal ATP-competitive kinase inhibitor staurosporine. Our data suggests that the causative genetic modification is likely a point mutation in the transcription factor CZT-1 that contains a Zn_2_Cys_6_ DNA-binding domain that binds to its target promoters, including genes involved in cell death and drug resistance. Noteworthy, the *acr-2* strain, which also displays increased resistance to acriflavine, harbors a gain-of-function point mutation on another Zn_2_Cys_6_ transcription factor (NCU05733) [19]. Zn_2_Cys_6_ proteins are fungal specific, constitute the largest family of transcription factors in *N. crassa* [22] and have been associated with drug resistance in various animal and plant pathogens [20,21,23]. Altogether, our data expand the role of CZT-1 as an important regulator of the fungal response to various drugs. In addition, the results show that acriflavine and staurosporine have overlapping intracellular targets; noteworthy, staurosporine is a protein kinase inhibitor [24] and acriflavine has been shown to inhibit the DNA-dependent protein kinase (DNA-PK) [25] and protein kinase C (PKC) [26]. Further studies will be needed to link these signaling transducers to their downstream transcriptional regulators during fungal cell death.
